# The Necessity for Wearing Glasses and a Plea for the Early Recognition of the Conditions Requiring Their Use

**Published:** 1888-11

**Authors:** W. H. Way

**Affiliations:** Atlanta, Ga., Ex-Resident Surgeon Manhattan Eye and Ear Hospital, New York


### THE NECESSITY FOR WEARING GLASSES AND A PLEA FOR THE EARLY RECOGNITION OF THE CONDITIONS REQUIRING THEIR USE.

BY W. H. WAY, M. D., ATLANTA, GA.,

Ex-Resident Surgeon Manhattan Eye and Ear Hospital, New York.

Of all of our senses, the sight is the one through which we receive the most of our pleasures, and the means by which we obtain our highest intellectual development. In truth, there is no organ in the body, the preservation of which in its integrity, adds so much to our enjoyment, and is so necessary to our future welfare, as the eye.

See then how it behooves us as physicians to be able to recognize at an early day those optical defects, which if allowed to go uncorrected, will limit the exercise of this valuable function, and perhaps entail much physical suffering upon the individual. When we come to consider what is required of a pair of eyes, it ceases to be a matter of wonder that we find so many that give way under the work required of them. From the time we awake in the morning to the hour of retiring at night this much-abused organ has been unremittingly at work. When we bear in mind that for all distances nearer than twenty feet there must be a certain co-ordination of the muscles of the eye, and a different curvature of the lens to enable us to see objects distinctly, we can imagine the amount of nervous innervation required, even in a normal eye, and with a perfect adjustment of the ocular muscles. Judge then what will be the effect should there be an error of refraction, or a lack of equilibrium.

In this struggle for preferment at the present day a far greater demand is made upon the eye than formerly. Take for example the education of the children. Although much has been done in the way of better ventilation, light, and hygienic surroundings, still much remains to be done yet in fewer hours spent in schoolrooms and less study at home. As they advance in years the work for the eyes increases in the anxiety to get through and begin life. Follow them as they are seen filling their various vocations, and you will find them using their eyes all day long—some far into the night, and in poorly lighted rooms, badly ventilated, and perhaps filled with tobacco smoke. Sooner or later the victims of such surroundings find themselves unable to keep up this continual strain, and they are to be found seeking advice in the consulting rooms of the specialist, and in the clinics of the eye and ear hospitals. Let us make a careful study of the clinical pictures which this numerous class of patients presents. In doing this we will find that there are three groups.

The first one and the largest consists of those who are called hypermetropes. Their troubles arise from a constant strain upon the ciliary muscle to produce such a curvature of the lens as will enable them to see all objects distinctly. This continuous use of the eyes brings about a number of reflex neuroses and symptoms, which for convenience's sake are termed asthenopia; prominent among which are headache, pain in the eyes, burning of same, blurring of vision, etc. Some of the objective symptoms are photophobia, lachrymation, conjunctivitis, blepharitis, hordeola, etc.

These patients seek advice because their eyes are in such a condition that they are unable to pursue their daily avocations without more or less discomfort.

There are children of whom the teachers complain of not being able to study long. Their lessons are imperfectly learned, and they are always more ready for play than books. They are the ones who finally suffer in their general health from various reflexes, are frequently dosed with drugs, taken from school, and thus lose much valuable time at a most important part of their life simply for the lack of a pair of glasses to correct their error of refraction. Among the adults are found book-keepers, draughtsmen, stenographers, those who use the needle, and in fact all whose occupations require the eyes to be constantly used at 12 to 18 inches.

Another group are the myopes, and they constitute a much smaller class than the one which we have just considered. Yet they too have their annoyances, and there exists great danger of serious consequences in the higher degrees if allowed to go uncorrected. The children are found among those who say that they cannot see the figures on the blackboard at school. They are usually quiet, and will spend most of their time indoors reading their books, and looking at the pictures, not because they are not fond of play, but they see so poorly in the distance that they become timid and will not run and romp like the other children. As they grow older they are known as the well-read young men and maidens, but this is all acquired at the expense of physical development in many instances. These latter now complain that they are not able to recognize their friends on the streets; they cannot read the signs or the numbers of the houses. The beauties of the stage are lost to them, and if not at an earlier date they now find that they cannot see as well as their friends. The condition of these patients is sometimes inherited or congenital. Rather often it is acquired; and in the higher degrees is progressive, and sometimes ends in the loss of all useful vision. It is a prolific source of hyalitis, choroiditis, and detachment of the retina. See then that this is a matter of the utmost importance, and that this class of patients should have their error of refraction corrected at the earliest possible day, and carefully instructed in the proper use of their eyes. It is the only way to enable them to preserve the usefulness of such vision as they may have. For the continued use of such eyes in the effort to obtain an education is frequently at the expense of their integrity.

Finally we come to the third and last group, and find them consisting of those who have passed forty years. With these it is simply a matter of inability to produce a certain curvature of the lens owing to its increased density. Some will say that they are compelled to hold their reading matter too far away; others that they have been obliged to give up reading at night; and the women will complain of not being able to thread their needles, or see how to do fine work. A few get alarmed and think that they are going to become blind. Experience has proved that the wearing of glasses with this class has now become a necessity, and that to delay any longer only deprives them of so much pleasure, and lessens their sphere of usefulness.

We have now taken a cursory view of the causes and conditions of a large number of patients who seek our advice because they are unable to pursue their occupations without discomfort. They belong to every age, sex, and condition of life, and are simply paying the penalty for being the "survival of the fittest." They are an integral part of our higher civilization, and they will always exist. They are here—what are we to do for them? Their education, their future success, and their families depend upon the continued use of their eyes. Are we to prescribe "eye drops," "eye washes," "eye salves," tonics, etc.? No; all they need is a pair of glasses to correct an ocular defect, and in the majority of cases the individual is at once restored to happiness and usefulness. A most important matter however, is the careful selection of these glasses, and how they are to be used.

Some are to be worn always; others only for doing near work; some for distant vision alone; and yet another class will require two pair, one for the distant, and another for the near. It requires no little training and experience for the practitioner to fill the requirements in these varied cases; and he who has not made himself familiar with this kind of work had better send his patient to one especially prepared for it. The specialist in this branch is peculiarly fitted for the correction of these errors of refraction, and no one can do the work intelligently or properly unless he has good light, plenty of space, and the apparatus necessary; neither can the work be done hurriedly. In many cases it takes a number of days to solve the difficulty. But it can be done, and those who have had the good fortune to have been directed to one possessed of such skill know the good results.

The correction of the errors of refraction is the drudgery of the practice of ophthalmology. But I know of work in no other field of labor that will relieve as many distressing neuroses, give more comfort to your patients, or yield more brilliant results. I know that there exists among the laity a great prejudice against the wearing of glasses, and no one whose eye troubles could possibly be relieved in any other way should be permitted to use them. Spectacles are to be worn to correct ocular defects, for the eye is an optical instrument as well as an organ; and when it can be demonstrated that their use enables one to see better or relieve them of eye strain, I doubt if there is any one who would not gladly accept them.

My object in writing this paper has been to portray in a plain and unvarnished manner a class of patients whose condition is the result of the intimate relationship between certain eye troubles and errors of refraction, and to show the necessity of an early recognition of the causes and the means of their relief.

*No. 48 Marietta street.*

